# Resting-State Functional Networks Correlate with Motor Performance in a Complex Visuomotor Task: An EEG Microstate Pilot Study on Healthy Individuals

**DOI:** 10.1007/s10548-022-00934-9

**Published:** 2022-12-25

**Authors:** Joaquin A. Penalver-Andres, Karin A. Buetler, Thomas Koenig, René M. Müri, Laura Marchal-Crespo

**Affiliations:** 1 Motor Learning and Neurorehabilitation Laboratory, ARTORG Center for Biomedical Engineering Research, University of Bern, Bern, Switzerland; 2 Psychosomatic Medicine, Department of Neurology, Inselspital, Bern University Hospital, University of Bern, Bern, Switzerland; 3 Translational Research Center, University Hospital of Psychiatry, University of Bern, Bern, Switzerland; 4 Perception and Eye Movement Laboratory, Department of Biomedical Research (DBMR) and Department of Neurology, Inselspital, Bern University Hospital, University of Bern, Bern, Switzerland; 5 Gerontechnology and Rehabilitation Group, ARTORG Center for Biomedical Engineering Research, University of Bern, Bern, Switzerland; 6 Department of Cognitive Robotics, Delft University of Technology, Delft, The Netherlands

**Keywords:** Resting-state, Functional connectivity, Electroencephalography (EEG), Microstates, Correlation, Motor performance

## Abstract

Developing motor and cognitive skills is needed to achieve expert (motor) performance or functional recovery from a neurological condition, e.g., after stroke. While extensive practice plays an essential role in the acquisition of good motor performance, it is still unknown whether certain person-specific traits may predetermine the rate of motor learning. In particular, learners’ functional brain organisation might play an important role in appropriately performing motor tasks. In this paper, we aimed to study how two critical cognitive brain networks—the Attention Network (AN) and the Default Mode Network (DMN)—affect the posterior motor performance in a complex visuomotor task: virtual surfing. We hypothesised that the preactivation of the AN would affect how participants divert their attention towards external stimuli, resulting in robust motor performance. Conversely, the excessive involvement of the DMN—linked to internally diverted attention and *mind-wandering*—would be detrimental for posterior motor performance. We extracted seven widely accepted microstates—representing participants mind states at rest—out of the Electroencephalography (EEG) resting-state recordings of 36 healthy volunteers, prior to execution of the virtual surfing task. By correlating neural biomarkers (microstates) and motor behavioural metrics, we confirmed that the preactivation of the posterior DMN was correlated with poor posterior performance in the motor task. However, we only found a non-significant association between AN preactivation and the posterior motor performance. In this EEG study, we propose the preactivation of the posterior DMN—imaged using EEG microstates—as a neural trait related to poor posterior motor performance. Our findings suggest that the role of the executive control system is to preserve an homeostasis between the AN and the DMN. Therefore, neurofeedback-based downregulation of DMN preactivation could help optimise motor training.

## Introduction

Humans go through a continuous process of acquiring new motor skills, from those required to meet fundamental needs such as ambulation and self-care, to more skilled movements including playing sports, music, and dancing. We might also encounter detrimental situations that demand us to relearn or circumvent lost function through intensive neurorehabilitation, e.g., after a brain injury. Given the impact on people’s lives, the topics of motor learning and relearning of lost functions have been extensively studied—see reviews in (Wulf 2013; Basalp et al 2021; Marchal-Crespo and Reinkensmeyer 2009; Sigrist et al 2013).

It is thought that motor learning and neurorehabilitation can be optimised by providing intensive functional movement training that promotes sensory input to the central neural system (Winstein et al 2014). Yet, not everybody learns equally when presented with the same task and training duration. Several factors have been observed to play a role in motor learning, e.g., the learners’ initial skill level (Basalp et al 2021; Ackerman 2007) or the learners’ approach to learning—e.g., individual learning strategies (King et al 2012). In biological terms, performance depends on individuals’ anatomical (Tomassini et al 2011) and functional brain organization (Raichlen et al 2016; Sugata et al 2020; Mary et al 2017), even before practice starts. Therefore, the understanding of how brain organisation correlates with motor behaviour may be a sound foundation to understand the differences observed between individuals.

The study of functional brain organisation is based on brain connectivity analyses between either different brain regions or different brain networks—i.e., a set of (typically distant) brain anatomical areas that exhibit a consistent global functional organisation, see reviews (He et al 2019; Deco and Corbetta 2011). Previous studies on healthy participants consistently showed that spontaneous (namely, resting-state) inter- and intra-areal functional brain connectivity before task execution is a robust predictor of both cognitive performance (Gui et al 2015; Schlee et al 2012; Wang et al 2010; Boly et al 2007), muscular fatigue (Li et al 2022), and motor performance (Raichlen et al 2016; Sugata et al 2020; Mary et al 2017; Faiman et al 2018). Resting-state functional connectivity has also been shown to predict motor and cognitive performance among stroke survivors (Dubovik et al 2012; Vicentini et al 2021; Hong et al 2019) and people suffering from Alzheimer’s disease (Cecchetti et al 2021; Dubovik et al 2013; Jones et al 2016, 2011).

In the context of motor learning, researchers found that increased connectivity between motor and sensory integration-related brain regions—e.g., involving the Primary Motor Cortex (M1) and the Parietal Cortex (PC) (Wu et al 2014; Manuel et al 2018; Berti et al 2019) or other areas belonging to the Sensorimotor Network (SMN) (Hong et al 2019; Carter et al 2010; Mottaz et al 2015) enhances motor performance. Conversely, researchers found that increased neural interactions between motor areas and areas related to attention or conciousness—e.g., M1 and the Teemporal Lobe (TL) (Sugata et al 2020), areas found in the Attention Network (AN) (Mary et al 2017), or regions within the Default Mode Network (DMN) (Raichlen et al 2016; Mary et al 2017)—is detrimental for motor performance.

While these findings rely on temporal correlations between pairs of preselected brain areas, the role of prior resting-state activity of global functional cognitive networks in posterior motor performance remains elusive. To address this literature gap, we propose the use of Electroencephalography (EEG) Microstate analysis—i.e., the identification of transient EEG periods (50–200 ms long) with stable spatial field configuration; see (Michel and Koenig 2018) for a review. Several research groups have established a correspondence between Functional Magnetic Resonance Imaging (fMRI)- and EEG-identified Resting-State Networks (RSN) named after the letters A to F (Britz et al 2010; Milz et al 2016). Unlike the techniques used in the articles mentioned above, the microstate corpus is based on the assumption that the overall pattern of interaction among brain regions is mediated by global and synchronous oscillations of cortical excitability that determine the functional state of the brain (Michel and Koenig 2018; He et al 2019).

Therefore, EEG microstates may be best suited to study the correlation between the preactivation of well-known, functional resting-state cognitive networks, and posterior motor performance. Other studies performing connectivity analyses based on electrode-based correlational studies focused on cognitive (Boly et al 2007), motor (Faiman et al 2018), or muscular (Li et al 2022) performance. In this study, EEG microstates allow to capture global brain states well-correlated with known functional cognitive networks and served to establish the correlation between the preactivation functional resting-state cognitive networks and posterior motor performance. Therefore, microstate analyses are complementary to other approaches. As a result, our findings extend the knowledge about the influence of cognitive brain states on motor performance beyond existing evidence linking muscular (Li et al 2022) and motor performance (Sugata et al 2020) to brain local activation patterns.

Previous research linked the appearance of DMN microstates—a predominant network in resting-state recordings (Kabbara et al 2021; Doucet et al 2012)—to different aspects of consciousness, cognitive control, self-referential thoughts, and mentation (Michel and Koenig 2018). For example, (Christoff et al 2016, 2009) attributed to areas such as Posterior Cingulate Cortex (PCC)—an area linked to the posterior DMN represented by microstates C and E (Britz et al 2010; Michel and Koenig 2018)—with mind-wandering. Importantly, people reporting self-reflected and internally-directed thoughts (mind-wandering) consistently show less robust performance during task execution (Christoff et al 2016). Further, robust performance is usually linked to lower movement variability. Concretely, internally directed focus of attention results in higher movement variability in motor tasks (Wulf et al 2001; McNevin et al 2003; Newell and Slifkin 1996) and deterred performance in tasks that require conscious, non-automatic, controlled execution (Smallwood and Schooler 2006). Researchers investigating resting-state neurophysiological activity prior to a golf swing found two prominent patterns of activation. First, novice players showed increased activity at the PCC, linked to difficulties in filtering out irrelevant information. Second, experienced players showed increased activity over areas related to the AN (Milton et al 2007). The involvement of the Inferior Parietal Lobe (IPL)—involved in the AN and imaged through Microstate D with sources in the parietal, frontal and insular cortex; (Custo et al 2017)—, is known to relate to lower movement variability during task execution (Haar et al 2017). Researchers found that the IPL may support participants’ attention towards task-relevant stimuli and sensorimotor integration during motor planning and execution (Wenderoth et al 2005).

In this study, we investigated whether prior mental states related to increased attention in the environment—represented by resting-state activity at the AN—and reduced mind-wandering— characterised by resting-state activity at the DMN network—are beneficial for motor performance in a complex visuomotor task: virtual surfing. Participants were asked to steer a virtual boat to surf waves as fast as possible towards a finish line (Penalver-Andres et al 2021). To accelerate on the wave, participants had to detect the incoming waves and align the boat towards the wave direction by turning a joystick. The resting state microstates were extracted during EEG recordings with closed eyes—e.g., as in (Michel and Koenig 2018)—and recordings with open eyes—e.g., as in (Deolindo et al 2021)—prior to the execution of the task. We hypothesised that:

1) The presence of Microstate D prior to motor execution—linked to better attention towards the external task-relevant wave onset— will be associated with low movement variability during task execution. We quantify movement variability as the standard deviation of the joystick turning angle.

2) The presence of Microstate C prior to motor execution will be correlated to poor motor performance, i.e., longer times to reach the finish line.

Our study serves to gain a better understanding of how resting-state networks affect posterior motor performance. We supplement previous research that was restricted to connectivity analyses between preselected pairs of brain areas. To the best of our knowledge, we are the first to study the relationship between network configurations at rest (i.e., microstates) and posterior motor performance. This understanding might help researchers consider prior participant-specific neural biomarkers to characterise participants’ neural traits and potentially design participant-specific motor training strategies, e.g., by using neurofeedback specifically-designed to attenuate or enhance activity of microstate networks known to correlate with specific aspects of motor performance (Mottaz et al 2015).

## Methods

The findings presented in this manuscript correspond to recordings conducted within a broader experiment. The experimental setup, motor task, and experimental protocol have previously been described elsewhere (Penalver-Andres et al 2021). In this document, only a short description is provided for completeness.

### Participants

We recruited 36 healthy participants who provided written consent to participate in the study. The study was approved by the Kantonale Ethikkommission Bern and the Swiss Agency for Therapeutic Products.

Our sample consisted of 14 women and 22 men, aged 20 to 59 years ($\mu _{age}$ = 27.9 years; $\sigma _{age}$ = 6.64 years). Despite the wide range of ages, significant brain structural changes are expected only among participants older than 35 years with a slight decline in brain tissue volume $0.2\%$ (Zanto and Gazzaley 2019; Hedman et al 2012). Changes in the functional network of the brain related to age are expected among participants in their 30s compared to participants in the 40s and 50s only at the Dorsal Attention Network and sensory/effector specific brain networks (e.g., auditory or hand networks, (Varangis et al 2019)). Thirty-one participants were right-handed according to the Edinburgh Handedness Inventory (Oldfield 1971). All participants were naive to the motor task. Several participants reported having some experience with virtual reality (13 participants), video gaming (14 participants), and sailing (4 participants, of which one had a sailing licence).

### Experimental Setup

**Fig. 1:**
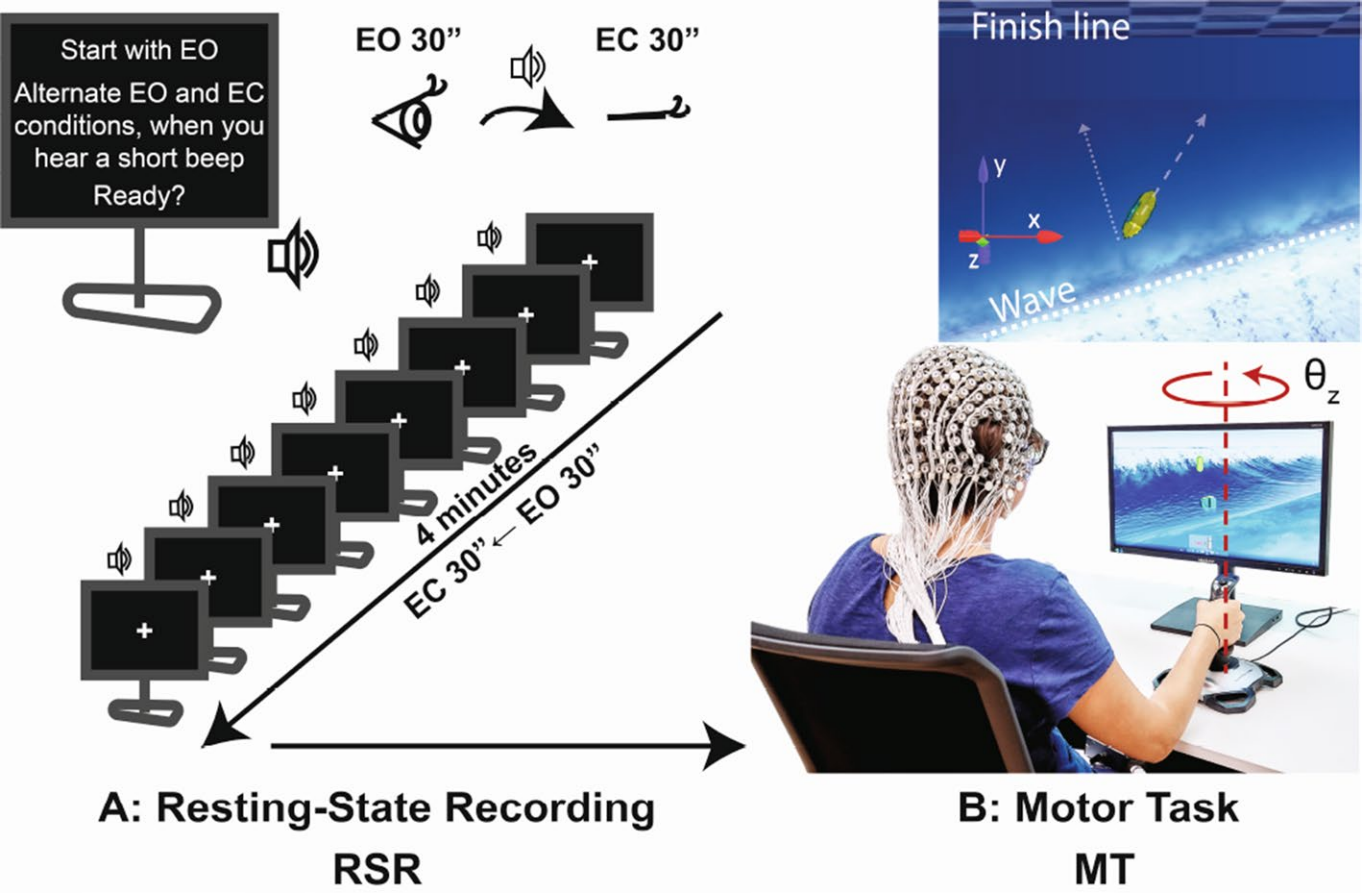
Protocol and experimental setup. **A** Resting-State Recording (RSR) protocol. Participants sat in a chair with chin rest, while wearing an EEG cap. During Resting-State Recording, they were facing a computer screen with a fixation cross in the middle while we recorded their electroencephalographic activity. Participants were asked to begin the Resting-State Recording with their *Eyes opened* (*EO*) and alternate with *Eyes closed* (*EC*), every 30 s. This sequence was repeated four times. Participants were asked to fixate their gaze on the fixation cross during EO and keep a *resting mind* during the whole resting-state recording. **B** Motor Task. After the Resting-State Recording, participants performed the Motor Task. They were instructed to steer a virtual boat on a wavy sea by turning a joystick and reaching the finish line as fast as possible. The boat accelerated when it was aligned with the wave direction

Participants performed a virtual surfing Motor Task (MT) developed in Unity (Unity Technologies, United States). Participants controlled the direction of a virtual boat by turning the vertical axis of a joystick (J-UK-17, Logitech, Switzerland) with their dominant hand (Fig. 1B). During the experiment, participants remained seated in a comfortable chair and rested their chin on a chin rest (not visible in Fig. 1B). The position of the joystick, the chair, and the chin rest was controlled across participants. We recorded the EEG activity of the participants using a 256-channel Hydrogel cap and EGI Net Amp amplifier (Electric Geodesics, United States).

### Resting-State Brain Activity Recordings

The experiment started with an EEG Resting-State Recording. Participants were asked to start the Resting-State Recording with the *Eyes Opened (EO)* and alternate with *Eyes Closed (EC)*, each block of 30s duration (Fig. 1A). This sequence was repeated four times—i.e., $4~x~(EO + EC) ~x~ 30~s = 4$ min. The start of each EO and EC block was indicated by a short beep presented through a loudspeaker mounted on the computer screen.

During the EO blocks, participants were requested to visually fixate on a white cross presented in the middle of the computer screen and *“stay awake and still”* (see exact instructions in Appendix). During the EC blocks, participants were requested to also *“stay awake and still”* but with their eyes closed. To avoid eye-related electroencephalographic artefacts, we asked participants to avoid, as much as possible, eye movements (e.g., eye blinks or lateral fixations).

Previous studies on resting-state microstate network identification have been performed with EC. This applies both to the identification of microstates based on EEG (Custo et al 2017), see (Michel and Koenig 2018) for a review. Yet, recent literature also included EO conditions in their experiments, e.g., (Deolindo et al 2021). Here, we focus on the analysis of the EC condition, but performed identical analyses with the data recorded during the EO condition to compare our findings between conditions (Barry et al 2007). Finally, we choose to alternate EC and EO conditions in order to avoid commonly found limitations of each experimental condition, observed during the piloting phase of this study: participants falling asleep, resulting in excessive alpha-band EEG signal presence (EC condition, (Berger 1929)) and participants experimenting dry eyes symptoms, including excessive blinking or tearing reflex (EO condition).

### Motor Task

After the Resting-State Recording, participants were requested to surf waves as fast as possible to a finish line in a virtual wavy sea [Motor Task (MT)]. The exactly written instructions were displayed on the monitor before the task started: *“You will surf with a boat on the sea. Surf as fast as possible to the finish line”*. The design of the motor task was described in detail in (Penalver-Andres et al 2021). Here, only a brief summary is provided for completeness.

To accelerate the virtual boat, participants had to discover and master an undisclosed underlying task rule: when a wave would reach the boat from behind (*incoming wave onset*), they had to align the boat direction with the direction of the wave by turning the joystick vertical axis (Fig. 1B). The wave direction changed among a pseudo-randomised pool of directions during the Motor Task. The virtual surfing task is a relatively complex visuomotor task that imposes high cognitive and motor demands on practitioners. Participants performing this task should pay attention to the timing and orientation of incoming waves. Then, they should execute swift and precise joystick turning movements with their hands to catch waves and accelerate the boat. Participants performed the Motor Task twice (4-5 min per task), each containing around 36 incoming waves.

### Data Processing and Statistical Analysis

#### Data Recording

The boat position and joystick turning angle ($\theta _z$; Fig. 1B) were recorded at $\sim 50~Hz$ in Unity. The data were then linearly resampled at 50 Hz. The EEG data were sampled at 1000 Hz, and timestamps were added to the EEG data each time participants had to open (EO) or close their eyes (EC).

#### Behavioural Metrics

Two different metrics were selected to evaluate the participants’ performance during the MT.

The *Completion Time (CT)* was computed as the elapsed time (in seconds) from the start of the MT until the boat reached the finish line. Low CT represents high performance in the MT.

The *Movement Variability (MV)* was computed as the standard deviation of the rotation around the joystick vertical axis ($\theta _z$, Fig. 1B). This metric quantifies the variability of the participants’ steering movements while surfing the waves. Low MV represents automaticity, which has been associated with external attention to stimuli relevant to the task. More automaticity is also characteristic of expert performance (Fitts 1967; Wulf et al 2001).

#### Resting-State Networks

*Preprocessing*: The EEG data were preprocessed offline following the so-called Makoto’s pipeline (Miyakoshi 2021), a procedure implemented in the Matlab-based toolbox EEGLab (Delorme and Makeig 2004) to preprocess the data in a semi-automated manner. We included 186 electrodes with a high signal-to-noise ratio in the preprocessing step, excluding electrodes at array boundaries heavily confounded with muscle artefacts in the neck, maxillary, mandibular, and eyebrow areas (see Appendix Fig. 4). An additional reason to avoid considering analysing those electrodes was the often experienced loss of skin contact of these electrodes. In other words, the EGI EEG Hydrogel cap did not reach the zygomatic, maxillary, sternocleidmastoideic, and occipital skin surfaces in most of the participants, and, therefore, we considered it to contain no signal of interest. Data were downsampled to 250 Hz and high-pass filtered (cut-off frequency 1 Hz). Line electrical noise was filtered out using pop_cleanline from EEGLab. Channels with nonphysiological artefacts (e.g., neck or mastoid muscle artefacts or eye movements) were interpolated using a spherical interpolation pop_interp from EEGLab. Following, common-average re-referencing was applied.

N Independent Components [ICs; (Delorme et al 2007)] were extracted from the EEG signal using runamica15 (Palmer 2015), being N the rank of the EEG signal covariance matrix. This was done to avoid linear dependency resulting from the common average reference (Miyakoshi 2021). The ICs were inspected and rejected, when applicable, following visual and semiautomated ICLabel-based (Pion-Tonachini et al 2019) supervised inspection. We rejected ICs whose probability of representing a brain source—as indicated by ICLabel—were below 40 % (Pion-Tonachini et al 2019).

Microstates’ topographic distribution and metrics (for example, duration, occurrence and contribution, a.k.a. coverage) have been found to be relatively consistent across many studies, regardless of the filtering approach, the number of electrodes, and the frequency band used (Khanna et al 2014; Férat et al 2022; Michel and Koenig 2018). Yet, to avoid muscular artefacts and non-physiological low-latency drifts (e.g., electrode-gel-skin contact), complying with most EEG-based microstate analysis paradigms (Michel and Koenig 2018), the preprocessed datasets were further band-pass filtered (low cut-off frequency of 2 Hz and high cut-off frequency of 20 Hz), before microstate extraction. We then segmented the clean EEG signals to obtain separate 2 min long datasets for each EC and EO condition—i.e., by concatenating 4 blocks of 30 s long EC and EO condition, respectively. During segmentation, the first and last 2 s of each 30 s block were removed from each dataset to avoid auditory electroencephalographic artefacts, which are linked to the primary auditory cortex reaction to the high pitch beep presented to the participants (Jung et al 1998). Note that, although muscle artefacts have been regressed out using IC, the removal of the first and last 2 s of each EO/EC condition further prevents the effect of potential muscle artefacts due to the high pitch beep indicating the switch between EC and EO conditions.

*Microstate Extraction*: All steps in the microstate extraction were conducted using the Microstates v1.2 EEG Lab toolbox. Microstates were identified in the individual datasets of both EC and EO conditions, using the k-means algorithm (Pascual-Marqui et al 1995) by using the Global Field Power peaks and seven classes (polarity invariant as explained in Michel and Koenig (2018)) with a maximum of five restarts. We chose seven classes to compare our microstates with those identified by Custo et al (2017). Further, the selection of seven components was confirmed by k-means clustering using the average explained variance, i.e., how much of the signal variability is explained by the fitted microstates, as recommended in (Michel and Koenig 2018). Using 6 microstates, the explained variance resulted in $76.8\pm 8.9\%$ and $78.8\pm 9.3\%$, for EO and EC, respectively. Using 7 microstates, the explained variance resulted in $78.3\pm 8.9\%$ and $80.0\pm 9.4\%$, for EO and EC, respectively. Using 8 microstates, the explained variance resulted in $78.3\pm 8.9\%$ and $80.2\pm 9.4\%$, for EO and EC, respectively. As the explained variance plateaued at 7 microstates, we evaluated whether combinations of some of the 8 microstates could resemble to any of the 7 microstates, as this is a known fact in the microstates field (Custo et al 2017; Michel and Koenig 2018). This was the case for microstates 1 and 3 (using 8 microstates) combining in microstate 4/D/AN of the finally selected Custo-like microstates. Therefore, we continued the rest of the analyses presented in this paper with the seven microstates presented in Appendix Fig. 5.

Following, we averaged condition-specific microstate maps, resulting in one grand-averaged microstate dataset per EC and EO condition. These grand-averaged microstate topographies were spatially correlated with the Custo et al (2017) maps to calculate the *Commonality* (C)—i.e., a quantitative assessment of the similarity between different microstate maps. The maximum value for the commonality is 1, indicating a high spatial correlation between two microstate maps. The commonality values of the grand-grand averaged datasets (i.e., the average of the grand-averaged maps of each EC and EO conditions) are also reported in Table 1. Finally, we report the commonality value (Table 1) and display the grand-averaged microstate topographies for EC and EO conditions side-by-side with the EEG identified microstate topographies reported in Custo et al (2017) (see Appendix Fig. 5A and B, respectively).

Additionally, to understand how much and how the normative resting-state networks were associated with posterior motor performance, the maps identified in Custo et al (2017) were fitted onto the individual EEG recordings of each condition per participant. The averaged explained variances for the EC and EO conditions were $76.4\pm 2.9\%$ and $74.4\pm 3.1\%$, respectively.

Several microstate metrics were computed (Michel and Koenig 2018):

- *Duration* The average time that a microstate was active, expressed in seconds (*s*).
- *Occurrence* The number of times that a microstate was active during each condition (EC/EO) of the Resting-State Recording. This is a unitless natural number.
- *Contribution* The time coverage of a microstate over the 2 min of each condition, expressed in percentage (%).

We chose the Contribution of a microstate as our primary EEG metric because this metric is a function of Duration and Occurrence over the total 2 min long duration of each EC and EO condition. Nevertheless, Duration and Occurrence metrics were inspected in order to understand whether our findings involving the Microstate Contribution were driven by the Duration or the Occurrence Microstate metrics.

**Fig. 2:**
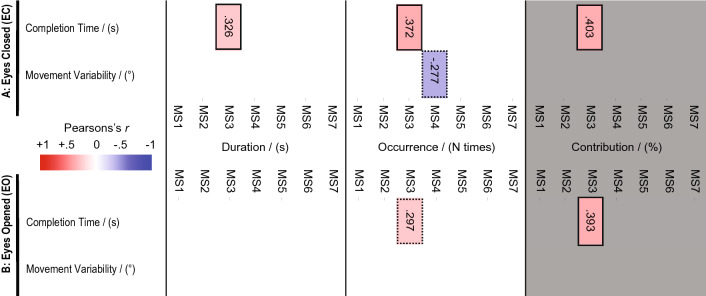
Correlations between behavioural metrics and resting-state network descriptors. Correlations between Completion Time, Movement Variability; and Duration, Occurrence and Contribution of each microstate resting-state network are displayed for Eyes Closed condition (EC, **A**) and Eyes Opened condition (EO, **B**). In grey shaded area, the correlations involving Contribution of a microstate (i.e.,primary EEG metric) are highlighted. Correlations with $.1\ge p > .05$ are boxed in dotted rectangles. Significant correlations with $p \le .05$ are boxed in full-line rectangles. Any other correlation resulted in $p > .1$ values, and thus, corresponds to blank matrix spaces. Pearson’s *r* correlation coefficients are displayed within the boxes and colour-coded in the figure

As a result, 21 microstate metrics (3 metrics x 7 microstates) were extracted per participant and condition (EC and EO). These 21 metrics were used for our correlational analyses, displayed in Fig. 2.

**Table 1:** Commonality values between Custo et al (2017) and Resting-State Recording microstates for each microstate and condition (i.e., EC and EO) and the grand-grand average of the EC and EO conditions

Microstate	1/A	2/B	3/C	4/D	5/E	6/F	7/G	$\mu \pm \sigma$
EC	0.973	0.932	0.974	0.929	0.820	0.954	0.963	$0.94\pm 0.05$
EO	0.982	0.916	0.943	0.963	0.900	0.970	0.969	$0.95\pm 0.03$
Grand-grand average	0.983	0.926	0.964	0.968	0.868	0.967	0.975	$0.95\pm 0.04$

The last column presents the average and standard deviation of the commonality values across all microstates

#### Statistical Analysis

**Fig. 3:**
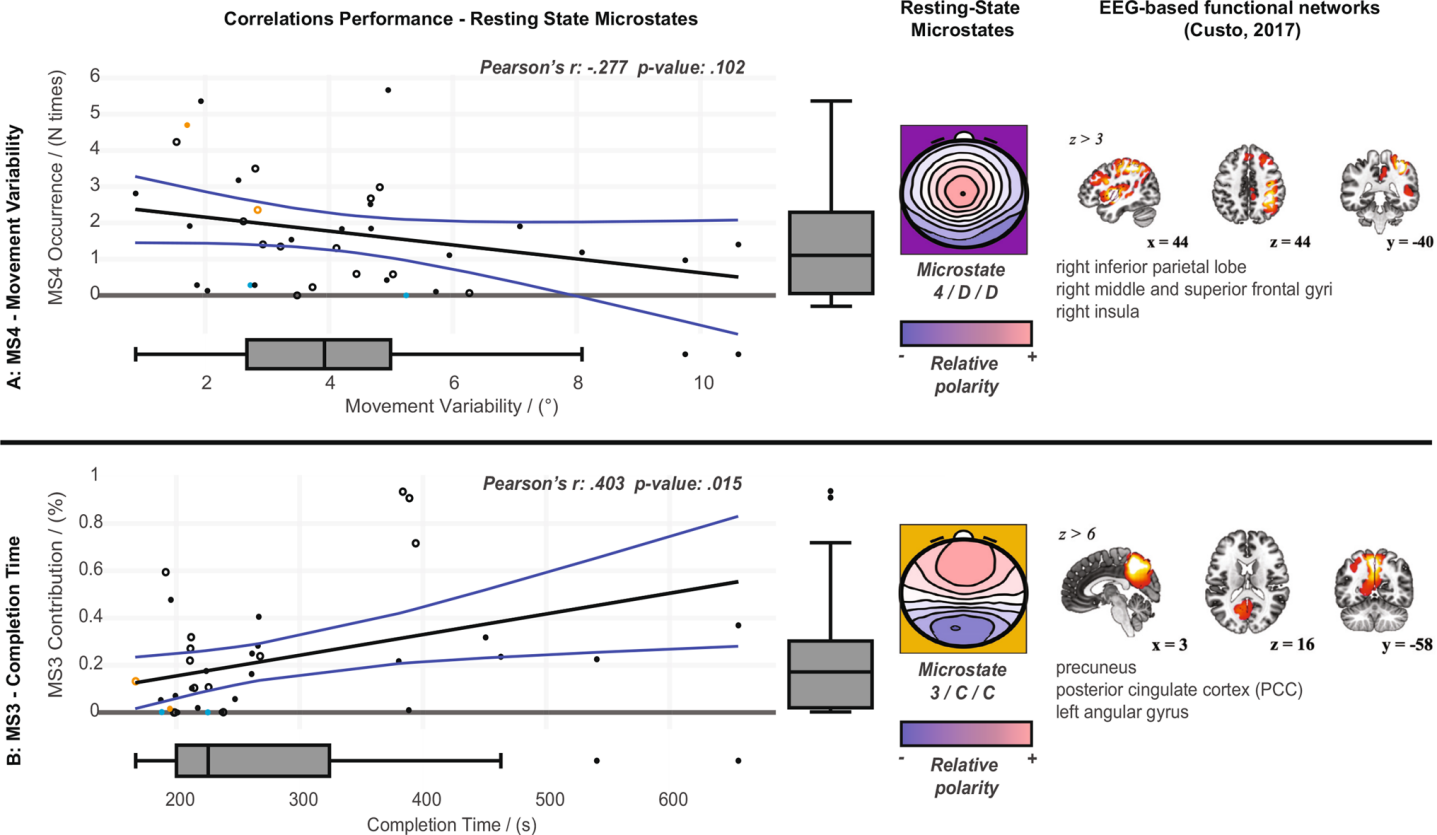
Correlations between movement variability (**A**) and completion time (**B**) and resting-state networks. *Left*: correlation between corresponding behavioural metrics and resting-state microstate metrics. Each dot represents one participant. Each empty dot is a participant with gaming experience. Each orange dot is a participant with sailing experience. Each blue dot is a participant with aged 40 or older (i.e., out of mean±standard deviation). Correlation lines indicate the best linear regressor line that fits the data (blue lines represent the confidence interval of the model fit). Pearson’s metric and *p*-values are indicated in each correlation plot. Boxplots on each figure axes describe the distributions of each variable: whiskers show the data ranging 1.5 times inter-quartile range above the upper or below lower quartiles. Boxed horizontal solid lines represent the median values and vertical box boundaries represent the inter-quartile range of the metric values. Data points out of the 1.5 inter-quartile boundaries are marked with dots. *Center*: EEG polarity-independent electrical scalp field corresponding to the microstates identified. Red to blue gradients (representing opposing polarities, with scaling corresponding to arbitrary units) are used to depict the polarity-independent voltage distributions corresponding to each microstate. *Right*: EEG-based functional resting-state networks represented by each microstate (images adapted from Custo et al (2017) with rights provided from Mary Ann Liebert, Inc.)

Pearson’s correlations were computed between each behavioural metric (i.e., CT, MV) and the 21 microstates metrics presented above. We found only significant (or tending to statistical significance) correlations between the behavioural metrics and several Microstate metrics of Microstates 4/D/AN and Microstate 3/C/posterior-DMN (see Fig. 2) . Nevertheless, to answer our hypotheses, we focus on correlational analyses between the Completion Time and the Contribution metric of Microstate 3/C/posterior-DMN [posterior-DMN (Custo et al 2017); Hypothesis 1] and between Movement Variability and the Contribution of Microstate 4/D/AN [AN (Custo et al 2017); Hypothesis 2] during EC see Figs. 2A and 3). For comparative purposes, we ran identical analyses involving the EO condition (Fig. 2B). Additionally, to understand whether our findings involving the Microstate Contribution were driven by the Duration or the Occurrence Microstate metrics, we visualised also existing correlations between behavioural metrics and Microstate Duration and Occurrence.

All found correlations with $p \le .10$ are displayed in a matrix fashion in Fig. 2. The correlations with $.1\ge p \ge .05$ are boxed in dotted rectangles. Significant correlation values ($p < .05$) are boxed in full-line rectangles. Values are presented for conditions EC and EO for comparative purposes. Finally, correlation plots investigating our hypotheses 1 and 2 involving exclusively EC Resting-State Networks are presented in Fig. 3. All microstate and behavioural metrics have been sanity-checked against potential confounding factors (e.g., participant’s age, gaming experience or sailing-experience). The results of these sanity-checks are reported in Sect. 3. To ease the visual inspection, the distribution of aged or sailing/gaming experienced participants are overlaid using a color/symbol code on Fig. 3.

### Data and Code Availability Statement

All results presented in this manuscript stemmed from data openly available in the following repository: 10.5281/zenodo.5883680

All results presented in this manuscript have been obtained by using publicly available toolboxes. For kinematics calculations the Python 3.7.1 and libraries Matplotlib, NumPy 1.15.4, pandas 0.23.4, and Scipy 1.1.0 were used. For microstate extraction the Microstate 1.2 toolbox within EEGLab 2019.0 was used. For statistics the toolboxes ggcorrplot 0.1.3, rstatix 0.7.0, sjstats 0.18.1 and readxl 1.3.1 were used.

## Results

### Commonality Between RSR and Custo et al (2017) Microstates

We observed that between the RSR-identified microstates and Custo et al (2017) maps, there was an average *commonality* of $0.95 \pm 0.04$ (taking together EC and EO) (EC: $\mu _{C}^{EC}=0.94, \sigma _{C}^{EC}= 0.05$; EO: $\mu _{C}^{EO}=0.95, \sigma _{C}^{EO}=0.03$). Detailed commonality values per condition and microstate are presented in Table 1.

### Correlational Analysis Between Preactivation of Microstate 4/D/AN and Robust Movement Execution

We did not observe a significant correlation between the *Movement Variability* and the Contribution metric of Microstate 4/D/AN ($r(34) = -.162, p = .347$). Additionally, we did not find a significant correlation between Movement Variability and Occurrence of Microstate 4/D/AN ($r(34) = -.277, p = .102$; Fig. 3A); nor between MV and the Duration of Microstate 4/D/AN ($r(34) = -.146, p = .396$).

### Correlational Analyses Between the Preactivation of Microstate 3/C/Posterior-DMN and MT Performance

Contribution of Microstate was positively correlated with *Completion Time* ($r(34) = .403, p = .015$; Fig. 3B). While the correlation between Completion Time and the Occurrence of Microstate 3/C/posterior-DMN was significant ($r(34) = .372, p = .026$), the Duration of Microstate 3/C/posterior-DMN showed only a trend towards a positive correlation with the Completion Time metric ($r(34) = .326, p = .052$) .

### Correlational Analyses Between Age, Sailing and Gaming Experience and Behavioural or Microstate Metrics

The age and sailing/gaming experience of participants is widely scattered throughout Fig. 3, relating performance and microstate metrics. The *Contribution and Duration of Microstate 7/F/Sensorimotor* positively correlated with age ($r(34) = .828, p < .001$ and $r(34) = .583, p < .001$, respectively). The *Contribution of Microstate 7/F/Sensorimotor* approached a negative correlation with participants’ gaming experience, but did not reach significance ($r(34) = -.285, p = .093$). The *Occurrence of Microstate 4/D/AN* positively correlated with participants’ sailing experience ($r(34) = .347, p = .038$). The *Occurrence of Microstate 3/C/posterior-DMN* approached a positive correlation with participants’ gaming experience, but did not reach significance ($r(34) = -.289, p = .087$). The *Occurrence of Microstate 3/C/posterior-DMN* negatively correlated with participants’ age ($r(34) = -.377, p = .023$) yet we must interpret this value with caution (see subgroup analyses excluding participants older than 40 y.o.). Neither participant’s age nor their gaiming experience correlated with *Movement Variability* nor *Completion Time*. Yet, both performance metrics approached significant negative correlations with the sailing experience ($r(34) = -.293, p = .083$ and $r(34) = -.324, p = .054$, respectively).

Finally, to further characterise the effect of age on our main results (presented in Fig. 3), we computed subgroup analyses based on Pearson’s correlations, excluding the two participants who depart from the median±std cohort’s age (i.e., aged older than 40 y.o.). In these analyses, we obtained similar trends but with lowered statistical power. The *Contribution of Microstate 3/C/posterior-DMN* positively correlated with the *Completion Time* ($r(32) = .384, p = .025$). The *Occurrence of Microstate 4/D/AN* negatively correlated with the *Movement Variability* ($r(32) = .286, p = .102$). Additionally, only the *Contribution and Duration of Microstate 7/F/Sensorimotor* positively correlated with age ($r(32) = .361, p = .036$ and $r(32) = .362, p = .036$, respectively). In the subgroup analyses, neither participants’ age nor their gaming experience correlated with *Movement Variability* or *Completion Time*, yet both performance metrics approached significant negative correlations with the sailing experience ($r(32) = -.302, p = .083$ and $r(32) = -.368, p = .032$, respectively). No other significant correlations with age, sailing or gaming experience were found.

### Comparison of Correlations Involving EO and EC Resting-State Microstates

During EO, the correlation between Completion Time of Microstate 4/D/AN and *Movement Variability* did not result statistically significant ($r(34) = -.163, p = .341$). Similarly, the correlation between Movement Variability and Occurrence of Microstate 4/D/AN ($r(34) = -.220, p = .197$) and Duration metrics of Microstate 4/D/AN ($r(34) = -.074, p = .669$) were not statistically significant.

Conversely, the previously reported correlations involving the posterior DMN and the Microstate resembled those observed in the EC condition (see Fig. 2B), at least in the Contribution metric. The Contribution of Microstate 3/C/posterior-DMN positively correlated with *Completion Time* ($r(34) = .393, p = .018$). The correlation between the Occurrence of Microstate 3/C/posterior-DMN and Completion Time did not reach statistical significance ($r(34) = .297, p = .078$). Finally, the correlation between Completion Time and Duration of Microstate 3/C/posterior-DMN did not reach significance either ($r(34) = .232, p = .174$), contrary to the significant correlation observed in the EC condition.

## Discussion

The study presented in this paper investigated the relationship between prior mental states related to attention in the environment and mind-wandering with posterior motor performance in a complex visuomotor task. We hypothesised that the preactivation of the Attention Network (AN) at rest (Microstate 4/D)—linked to enhanced attention towards external task-relevant stimuli (Wenderoth et al 2005)—would correlate with low movement variability (Milton et al 2007), and thus, result in more robust motor execution (Wulf 2013; Haar et al 2017). We also hypothesised that the preactivation of the Default Mode Network at rest—linked to mind-wandering—would be negatively associated with motor performance (i.e., completion time). This hypothesis was based on previous literature that showed that resting-state activity involving the DMN was negatively associated with motor performance (Sugata et al 2020; Milton et al 2007).

### The Posterior DMN Deters and the AN May Enhance Motor Performance

Previous literature has reported an anticorrelation between the AN and the DMN networks (Chang et al 2013; Raichlen et al 2016). In both motor and cognitive domains, complementary connectivity patterns between the DMN and the Sensorimotor Network (Mary et al 2017) or the AN (Maillet et al 2019; Gao et al 2019) seem to represent, respectively, externally versus internally diverted attention.

On the one hand, attention towards external task-relevant stimuli is thought to facilitate robust motor execution characterised by low motor variability (Wulf et al 2001; Lewthwaite and Wulf 2017; Wulf 2013; Newell and Slifkin 1996; Wenderoth et al 2005). Compared to an internal focus of attention, the participant’s external AN-mediated focus of attention results in enhanced motor performance thanks to high frequency-low amplitude adjustments (Wulf et al 2001) driven by enforced central nervous system inhibition mechanisms (Kuhn et al 2017; Wenderoth et al 2005).

On the other hand, the preactivation of the posterior DMN at rest has been linked to internally oriented self-referential thoughts (Christoff et al 2009), which seem to negatively impact not only motor but also cognitive performance (Smallwood and Schooler 2006). In fact, previous fMRI studies also pointed towards a negative effect of the DMN on motor performance. Findings from Berti et al (2019) suggest that shorter reaction times in Karate punches are negatively correlated with the connectivity between dorsal AN and posterior parts of the DMN (i.e., precuneus). Milton et al (2007) showed that, among other areas, novice golfers activate the Posterior Cingulate Cortex significantly more often than expert players when performing motor imagery of a golf swing movement. The authors linked the hyperactivation of the posterior DMN with a lack of skills to filter task-irrelevant stimuli among novice trainees. Additionally, Puttemans et al (2005) found a decrease in Posterior Cingulate Cortex activation when participants showed high levels of automaticity in a bimanual wrist synchronisation task. Yet, we would like to emphasize that during resting-state and especially mind-wandering, the DMN likely represents the most prominently active network (Kabbara et al 2021). Therefore, our results may relate to a relatively higher contribution of the posterior DMN, instead of other less-active competing networks, e.g., the AN or the Sensorimotor Network, which in turn resulted in poorer motor performance.

All in all, previous findings suggest that task-specific attentional focus is AN-driven but DMN-hampered. Therefore, we expected that the preactivation of the posterior DMN—linked to internally directed focus of attention—would be detrimental for motor performance. Conversely, the preactivation of the AN—linked to externally directed focus of attention—would be linked to enhanced motor performance.

However, contrary to our expectations, the presence of the AN at rest—previously linked to Microstate 4/D/AN (Britz et al 2010; Milz et al 2017; Michel and Koenig 2018; Custo et al 2017)—did not show a significant association with any behavioural metric. Only a nonsignificant correlation between the Occurrence of Microstate 4/D/AN and the Movement Variability was found. However, we observed that participants who showed a higher Contribution of Microstate 3/C/posterior-DMN—linked to the posterior DMN—at rest displayed worse performance in the following Motor Task as observed in a longer time to reach the finish line. Therefore, our findings only confirm our second hypothesis: the presence of Microstate C/posterior DMN during resting-state recordings prior to Motor Task leads to poorer motor performance during the complex visuomotor virtual surfing paradigm.

Previous studies showed that the relative alpha power negatively correlated with microstates occurrence (Khanna et al 2014). Therefore, the found lower occurrences of Microstate 4/D/AN could correspond to higher alpha-band activity over the scalp. This finding has been previously linked, especially in regards to the Visuo-attentional Dorsal Attention Network, with traits of increased performance (Penalver-Andres et al 2021). However, based on the limited power of our findings regarding Microstate 4/D/AN, we advise caution when interpreting our findings. Future studies are needed to investigate the relationship between different frequency bands and microstates.

To the best of our knowledge, our findings demonstrate for the first time in an EEG study the negative influence of the posterior DMN on motor performance. Previous research presents consistent patterns of activation in *on-task* attention networks and corresponding deactivation in the Default Mode Network, when attention is oriented toward the external environment (Raichle 2015). With the due caution, the opposite patterns of correlation of the AN and the DMN with motor performance found in our study could point towards an homeostatic mechanism that regulates internally versus externally directed attention (Milton et al 2007; Raichle et al 2001).

The loss of homeostasis between activation of the AN—which shares resources with the Central Executive Network (Vincent et al 2008)— and the DMN seems to be behind the pathophysiology of diverse attentional disorders—e.g., ADHD (Sudre et al 2017)—or reality detached statuses—e.g., schizophrenia (Michel and Koenig 2018; Luo et al 2020)). Researchers argue that frontal networks related to executive control (e.g., the ACC, superior frontal gyrus) would be acting as an *orchestra master* of the AN and the DMN (Vincent et al 2008). Despite we had no specific hypotheses about the role of frontal executive control networks for this study, they might underlie the regulation of internally versus externally directed attention, which subsequently affected performance in our study.

### Microstates are Robust Resting-State Biomarkers of Functional Cognitive Traits

In this study, we observed microstates at rest which share high commonality with those observed in previous studies Custo et al (2017). This holds for both Eyes Closed and Eyes Opened recordings, following a conventional resting-state microstate extraction pipeline.

Likewise, our findings link performance and Resting-State Networks with similar trends across Eyes Closed and Eyes Opened conditions. Other studies have found similar consistent DMN activation patterns during Eyes Closed and Eyes Opened rest across different EEG and Magnetoencephalography datasets following different processing pipelines (Kabbara et al 2021).

Previous research indicates that microstates can be used to image changes of functional mind states, e.g., whether participants showed task-dependent AN activation. In the experiment of Milz et al (2016), the authors observed a decrease in Microstate D in attentional tasks compared to rest. Paradoxically, Seitzman et al (2017) observed that Microstate D was enhanced during an attention-demanding task, compared to rest. On the other hand, during sustained attention tasks, the DMN activation is common during mind-wandering periods linked to higher reaction times, compared to *on-task* attention (Zanesco et al 2020).

While generally studies investigating the AN involvement were relative to a rest condition (Seitzman et al 2017; Milz et al 2016)—and, thus, affected by the exposure to the task goals—, the resting-state recordings reported here were performed before Motor Task and, thus, our participants were fully naïve to the task. Therefore, we propose that our findings do not reflect the effect of (transient) mind states on posterior motor performance, but (permanent) trait-like resting-state network preactivation correlated with later motor performance.

Microstates have been used to study neural traits related to, for example, Alzheimer’s disease (Smailovic et al 2019) or healthy aging research (Jabès et al 2021). Further, studies have focused on quantifying local connectivity patterns between pairs of motor and attentional or DMN areas to predict performance improvements or deterioration, respectively. In contrast, our study is, to the best of our knowledge, the first to show that trait-like global resting-state network preactivation correlates with posterior motor performance (particularly the preactivation of the posterior DMN). (Mary et al 2017; Berti et al 2019; Mattar et al 2018).

### Study Limitations and Research Opportunities

One of the main limitations of our study is the low statistical power of our analyses. For the Microstate 3/C/posterior-DMN, with Pearson’s $r = .403$ (moderate correlation, (Sullivan and Feinn 2012)) we found a coefficient of determination of $\rho ^2 = .1624$. For the Microstate 4D/C/AN, with Pearson’s $r = .277$ (small correlation), we found a coefficient of determination of $\rho ^2 = .0767$. Therefore, for a two-tailed hypothesis testing, we estimated that the power ($1-\beta$, being $\beta$ the type II error rate) to estimate any correlation different than Pearson’s $r = .0$ (null-hypothesis) is .7016 for the case of Microstate 3/C/posterior-DMN (.8026, if one-tailed) and .3789, for Microstate 4/D/AN (.5055, if one-tailed). Despite that the correlation effect found between the resting-state Contribution of Microstate 3/C/posterior-DMN and the Completion time yields accceptable power against type II error, we estimated that we would have needed over 100 participants to confirm our hypothesis for the relation between Microstate 4/D/AN and Movement Variability. Computations were conducted assuming a two-tailed Bivariate normal model in GPower v.3.1.9. (Faul et al 2009). Therefore, larger sample size studies would be necessary to confirm our integrative interpretation in the framework of motor learning. We evaluated whether our dataset could be fitted with other models (e.g., exponential models, see Appendix Fig. 6). However, we did not find another model that outperformed the linear fit, in line with previous studies (Mary et al 2017; Sugata et al 2020). Furthermore, our study can only make a statement relating to motor performance and not learning (i.e., long-lasting performance changes). To extend our findings to motor learning applications (Sugata et al 2020; Mary et al 2017) studies including retention tests are recommended.

The duration of the alternating Eyes Closed and Eyes Opened conditions was set to 30 s in our study. Previous studies investigating, for example, spectral differences between EO and EC conditions (Barry et al 2007) have used alternating sequences of 2 min. Faced with the trade-off of choosing the duration of each condition, we decided to mitigate the effects of eye dryness (during EO) and drowsiness (during EC) at the cost of having shorter condition-specific phases. Yet, the type of analyses we used (i.e., microstates) has a temporal resolution that allows capturing enough information in 30 s, i.e., the typical microstate duration ranges between 60–120 ms and an average occurrence between two and four times per second (Koenig et al 2002). However, the alternating protocol could potentially enhance attention during resting state compared with longer non-alternating intervals. One potential improvement to our paradigm would consist of using longer EC recordings where participants are reminded to stay awake with a gentle sound that can be back-traced to the EEG recordings to correct resulting potential artefacts.

Additionally, interrogating participants about their thoughts during Resting-State Recordings—namely, experience sampling techniques (Christoff et al 2009)—, would help characterise the participants’ potential mind-wandering experiences. Our study included two participants that were older than 40 years old. Therefore, ageing effects could have influenced our results. Significant brain structural changes are mostly expected among participants older than 35 years, with a slight decline in brain tissue volume of $0.2\%$ (Zanto and Gazzaley 2019; Hedman et al 2012). Changes in the functional network of the brain related to age are expected among participants in their 30 s compared to participants in their 40 s and 50 s, mostly at the Dorsal Attention Network and sensory/effector specific brain networks (e.g., auditory or hand networks (Varangis et al 2019)). To address this limitation we performed supplementary analyses to further characterise the effect of age on our main results correlation analyses with and without the older subjects (namely, subgroup analyses). In the subgroup analyses we obtained similar trends as in the original analyses (including eldest participants), yet, with lowered statistical power (due to the decreased number of data points). The Contribution and Duration of Microstate 7/F/Sensorimotor positively correlated with age, perhaps related to findings reported in (Varangis et al 2019). Nonetheless, no other significant correlations of neurophysiological or behavioural metrics were found with age, sailing or gaming experience. Therefore, even though we could show that age and gaming/sailing experience factors are unlikely driving our main findings, it would be informative to stratify for several confounding factors to expand beyond the findings reported in this pilot study with larger sample size studies.

It is important to note that the microstate extraction process relies on a mathematical representation of the brain global activity as a temporal succession of most-probably active brain networks—see mention to “winner-takes-all” clustering approach in (Michel and Koenig 2018). Therefore, despite our results showing a specificity towards Microstate 3/C/posterior-DMN and Microstate 4/D/AN, this does not mean that the other networks were not active during our resting-state recordings. Finally, our findings are certainly specific to the performance metrics selected and restricted to the seven microstates commonly used in the literature (Custo et al 2017; Michel and Koenig 2018), but it is possible that other networks might contribute to different aspects of motor performance. Furthermore, comparisons of resting-state microstates before and after practise, or between rest and task execution, would be needed to characterise the permanence of our trait-like findings and to assess whether these networks are also informative of transient mind state changes (Milz et al 2016; Seitzman et al 2017).

Finally, we chose to base our work on the findings of Custo and colleagues, instead of estimating our own source-level networks. Considering the volume conduction effect of EEG, similar scalp distribution might be contributed by different source activities (He et al 2019). Also, identical inverse solutions, estimated from scalp-level voltage distributions, may result from different source activities (He et al 2019). Furthermore, estimating resting-state sources from EEG resting-state recordings have proven to be a complex endeavour because of the low SNR present in the signal (Custo et al 2014). In order to perform similar analyses to (Custo et al 2014, 2017), we would need a longer resting-state recording of ideally a single condition (e.g., eyes closed) to be able to regress out enough signal to identify significantly-different sources that related to microstate maps. As this was out of the scope of this work, we decided to base our discussion on the source-level networks identified in a bigger cohort study (Custo et al 2017). While the topographies of functional microstates are similar across studies and have been often reproduced in literature (Milz et al 2017; Britz et al 2010; Custo et al 2017), conclusions on the underlying brain networks have to be interpreted with caution, as many brain areas contribute to a given microstate topography. Future studies could use longer resting-state recordings and bigger samples in order to perform source-level analyses using the Topographic Electrophysiological State Source-imaging (TESS) proposed by (Custo et al 2014).

Besides the study limitations, the method proposed in this study presents several relevant methodological points worth discussing. First, we make here a statement about Resting-State Networks and not about connectivity between preselected pairs of brain areas (Manuel et al 2018; Sugata et al 2020; Mary et al 2017). Microstates image long-range synchronous oscillations that determine functional states of the brain. Therefore, we talk about functional networks. Second, our findings could be applied beyond a predictive setting (i.e., using neural traits to infer posterior motor performance), to support diagnostic or even prescriptive applications. More frequent resting-state recordings interleaved with motor practise could help to detect abnormal activation patterns of the DMN, and to restore the balance with its counterpart, the AN, in order to ensure correct performance during a motor task or training. For example, microstate-informed neurofeedback could be a way of acting upon faulty DMN traits (Ge et al 2015; Pamplona et al 2020; Garrison et al 2021; Marins et al 2019; Rubia et al 2019; Diaz Hernandez et al 2016).

## Conclusion

To our knowledge, this is the first EEG study to analyse the effects of two prominent resting-state networks (i.e., the Attention Network and the Default Mode Network) on posterior performance in a complex visuomotor task: virtual surfing Motor Task. With the findings of this study, we confirm that the preactivation of the posterior Default Mode Network negatively correlates with posterior performance during our Motor Task. In conjunction with previous research, our findings confirm that mind-wandering Default Mode Network networks might be detrimental to motor performance. Generally, using this approach, researchers could identify participant-specific neural traits—associated to well-known functional cognitive networks—correlated with specific motor performance aspects, to identify causes of, e.g., poor motor performance linked to Default Mode Network preactivation. Using techniques such as neurofeedback, the activity of networks linked to poor motor performance (e.g., the Default Mode Network) could be down-regulated prior to motor practice.

## Figures and Tables

**Fig. 4:**
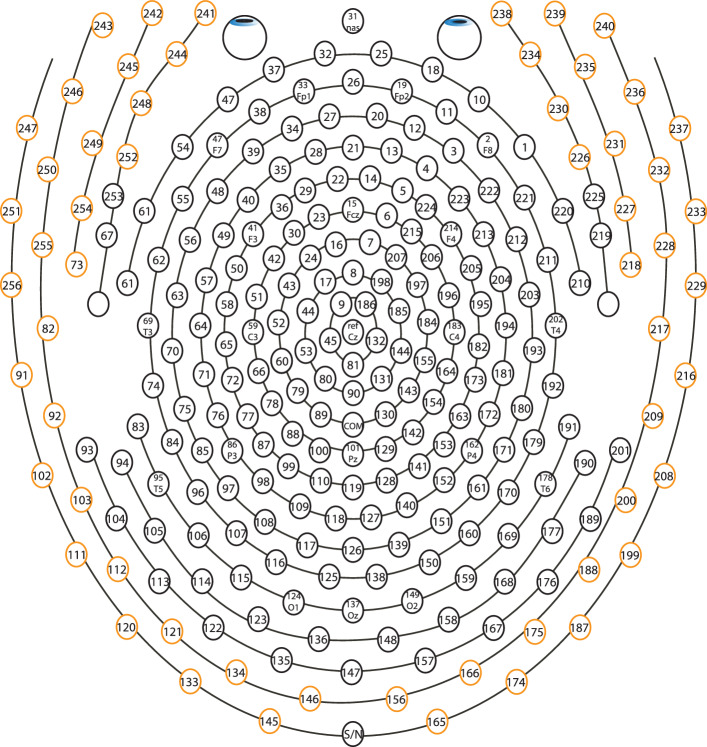
Electrodes discarded from the original EEG cap configuration. We used the 256-channel Hydrogel cap and EGI Net Amps amplifier (Electric Geodesics, United States). From the 256, only 199 electrodes were preserved. Electrodes marked in orange were discarded due to low signal-to-noise ratio

**Fig. 5:**
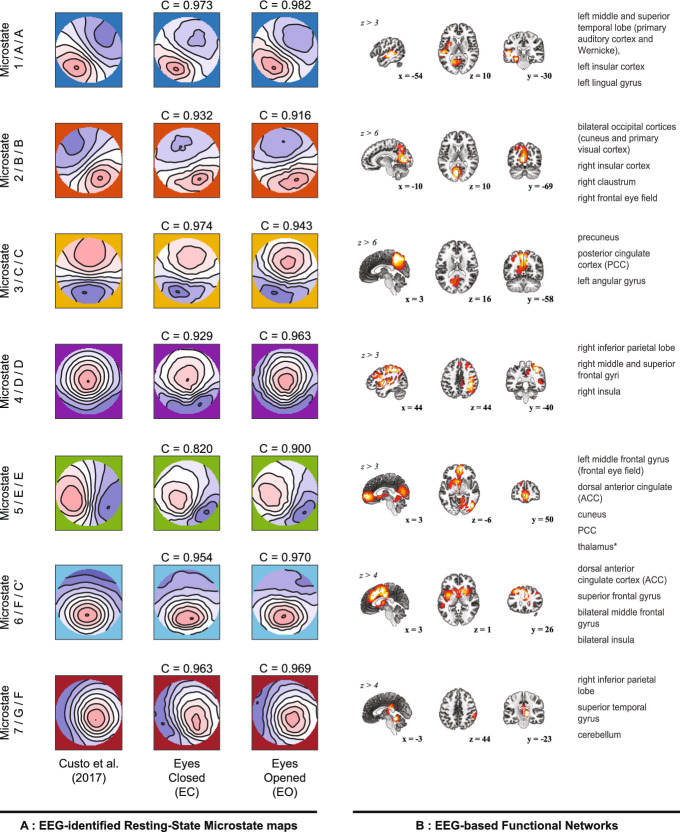
EEG-based Resting-State Networks (**A**) and corresponding Functional Resting-State Networks (**B**) The EEG-identified polarity-independent Resting-State Networks (**A**) show the equivalent topographical distribution of the electrical scalp field potential for each microstate corresponding to Custo et al (2017) and the Eyes Closed (EC) and Eyes Opened (EO) conditions in this study. Microstates are named after either a number (microstates computed in this study) or letters (the first letter indicates the corresponding microstate in Custo et al (2017) and the second letter represents the corresponding maps in Michel and Koenig (2018). Red to blue gradients are used to depict the voltage distribution corresponding to each microstate. Commonality values (C) w.r.t. the Custo et al (2017) maps are printed on top of each map. The EEG-based Functional Resting-State Networks (**B**) corresponding to each microstate are depicted, including sagital (left column), coronal (central column) and frontal (right column) sliced views. MNI coordinates are displayed on each slice. Areas of interest involved in each microstate-related network are listed in the right-most column of B (images adapted from Custo et al (2017) with rights provided from Mary Ann Liebert, Inc.)

**Fig. 6:**
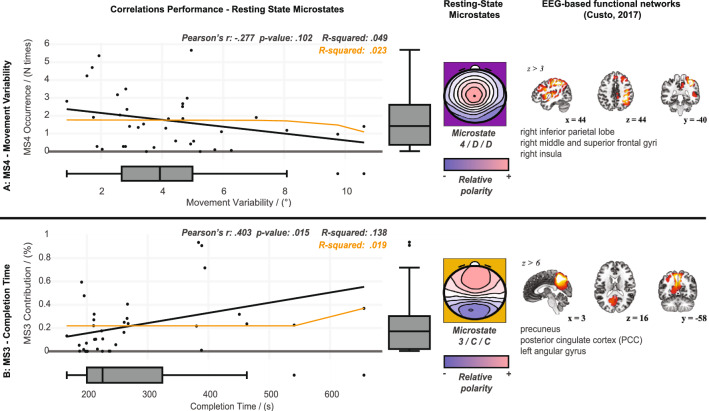
Relationship between movement variability (**A**) and Completion time (**B**) and resting-state networks. *Left* Relation between corresponding behavioural metrics and resting-state microstate metrics. Each dot represents one participant. Regression lines indicate the best model regressor line that fits the data. Pearson’s metric and *p*-values are indicated for linear regressors, in each correlation plot. Color coded R-squared values are printed for each regression model (black: linear regression, orange: exponential regression). Boxplots on each figure axes describe the distributions of each variable: whiskers show the data ranging 1.5 times inter-quartile range above the upper or below lower quartiles. Boxed horizontal solid lines represent the median values and vertical box boundaries represent the inter-quartile range of the metric values. Data points out of the 1.5 inter-quartile boundaries are marked with dots. *Center:* EEG polarity-independent electrical scalp field corresponding to the microstates identified. Red to blue gradients (representing opposing polarities, with scaling corresponding to arbitrary units) are used to depict the polarity-independent voltage distributions corresponding to each microstate. *Right:* EEG-based identified functional resting-state networks represented by each microstate (images adapted from Custo et al (2017) with rights provided from Mary Ann Liebert, Inc.)

## Appendix 1: Instruction

You will start with *eyes open* after the first “long beep”. After each “short beep”, change from eyes opened to eyes closed and viceversa. Every time you have your eyes open look at the white cross you see in the middle. In the end you will here a “long beep” to close the exercise.

## Appendix 2: EEG Cap Electrodes Discarded

See Fig. 4.

## Appendix 3: Microstate Map Correspondence with EEG-Based Functional Networks in Custo et al (2017)

See Fig. 5.

## Appendix 4: Different Fitted Models Representing the Relation Between Behavioural Metrics and Resting-State Networks Descriptors

See Fig. 6.
